# Integrated Multi-Omics Analysis Reveals Dysregulated Lipid Metabolism as a Novel Mechanism in Androgenetic Alopecia

**DOI:** 10.3390/biomedicines14010160

**Published:** 2026-01-12

**Authors:** Xiao-Shuang Yang, Liyang Duan, Yu-Jie Miao, Zhongfa Lu, Ru Dai

**Affiliations:** Department of Dermatology, Second Affiliated Hospital of Zhejiang University School of Medicine, Hangzhou 310009, China3200105360@zju.edu.cn (L.D.); 22118624@zju.edu.cn (Y.-J.M.)

**Keywords:** androgenetic alopecia, lipid metabolism, hair follicle, ACAA1

## Abstract

**Background and Aims:** Androgenetic alopecia (AGA) represents the most prevalent multifactorial condition leading to hair loss, necessitating an enhanced molecular understanding. The aim of this study is to present the analysis integrating protein, mRNA and miRNA between frontal and occipital regions of patients with androgenetic alopecia (AGA) and to identify potential mechanism. **Methods and Results:** Paired frontal and occipital scalps from four male donors with AGA were collected for transcriptomic and proteomics analyses. The molecular and protein characteristics of AGA were demonstrated by a comprehensive bioinformatics approach. Additionally, immunofluorescence (IF) and dual-luciferase reporter (DLR) assays were employed to confirm the analytical findings. A total of 758 differentially expressed proteins (DEPs), 1802 differentially expressed mRNAs (DERs) and 61 differentially expressed miRNAs (DEmiRNAs) were identified. Gene Ontology (GO) and Kyoto Encyclopedia of Genes and Genomes (KEGG) pathway analyses revealed significant enrichments in lipid metabolism, especially those involving PPAR signaling. Co-expression analyses further supported the association of up-regulated genes with lipid metabolism. A protein–protein interaction network analysis, supplemented by KEGG enrichment and the MCE algorithm, pinpointed four candidate genes: *DBI*, *ACAA1*, *IDH1* and *PEX3*. IF confirmed significant upregulation of ACAA1 and PEX3 in scalp tissues with AGA, while IDH1 was downregulated and DBI without significant changes. A competing endogenous RNA network indicated that hsa-miR-1343-3p targets *ACAA1* and hsa-miR-3609_R-2 targets *IDH1*, which were confirmed by DLR assays. **Conclusions:** This study provides preliminary evidence that hsa-miR-1343-3p-mediated regulation of *ACAA1* contributes to AGA pathogenesis, suggesting a link between AGA and lipid metabolism.

## 1. Introduction

Androgenetic alopecia (AGA) is the most common form of hair loss worldwide, characterized by progressive hair miniaturization and shedding, primarily affecting the frontal and vertex scalp. The primary pathogenic mechanism involves dihydrotestosterone (DHT) binding to androgen receptors in dermal papilla cells, leading to hair follicle miniaturization and premature entry into the telogen phase [1]. Clinical practice has shown that blocking androgens does not consistently reverse follicular miniaturization. For the frontal area, the efficacy of one-year oral finasteride treatment is approximately 20% [2]. Additional factors, including genetic predisposition, disorders of lipid metabolism, chronic inflammation, and oxidative stress, are believed to contribute to AGA onset and progression [3]. A growing body of evidence links AGA with significant comorbidities, such as hypertension, hyperinsulinemia, and obesity, suggesting a shared underlying biological mechanism [4].

Lipids, small molecules serving as both energy sources and signaling molecules, are also essential components of cellular membranes. Lipid metabolism, a process critical in various diseases including metabolic syndrome (MetS), tumors, and coronary heart disease [5], encompasses the synthesis, degradation, and transformation of intracellular fats. These processes are essential for sustaining vital physiological functions, such as maintaining membrane structure, providing energy, and facilitating signal transduction [6,7]. Furthermore, cholesterol and lipid metabolism are vital to hair follicle biology [8].

A robust correlation has been established between AGA and lipid metabolism. Individuals with AGA are more susceptible to developing metabolic syndrome, exhibiting abnormal lipid profiles, and experiencing specific alterations in lipid metabolites [9,10,11]. A meta-analysis showed that the risk of metabolic syndrome in AGA patients was 3.46 times higher than in controls (95% CI: 2.38–5.05) [10]. A meta-analysis of 19 observational studies (2561 AGA patients and 2098 healthy controls) showed that serum total cholesterol, triglyceride (TG), and low density lipoprotein (LDL) cholesterol levels were significantly higher in the AGA group than in controls [11]. Furthermore, a lipidomic study by Shuqin Wang et al. identified 51 differentially expressed lipids in the serum of AGA patients (*n* = 85) compared to healthy controls (*n* = 84). Among these, TG (15:0/18:1/18:1), phosphatidylcholine (PC) (16:2/21:6), and PC (43:6E) were considered potential biomarkers for male AGA. The study also found that certain specific lipids in AGA patients, based on their altered levels, were significantly correlated with DHT, prostaglandin D2 (PGD2), and TGF-β1, as well as markers of metabolic syndrome [9].

In this study, we elucidate the potential role of lipid metabolism in AGA through multi-level analyses of proteins, mRNA, and microRNAs.

## 2. Methods

### 2.1. Clinical Sample

This research was carried out at the Second Affiliated Hospital of Zhejiang University School of Medicine in strict adherence to the Helsinki Declaration’s ethical standards. Informed consent was secured from all study participants, and the protocol was sanctioned by the hospital’s Ethics Committee (IRB20230380). The study included seven male subjects, aged between 29 and 46, who were diagnosed with Hamilton-Norwood Class III-V AGA. They were recruited from June to December 2023, and diagnosis was verified by a specialist in alopecia. All enrolled patients had no history of chronic diseases requiring long-term medication (e.g., hypertension, diabetes) or endocrine disorders. Furthermore, none of the patients had taken any medications known to potentially affect hair growth or lipid metabolism (including, but not limited to, finasteride, dutasteride, minoxidil, spironolactone, and oral or topical glucocorticoids) for at least six months prior to enrollment. Additionally, patients had not undergone any specific dietary interventions or used medicinal hair care products that might interfere with local scalp metabolism prior to sample collection. We collected paired biopsies from the alopecic frontal region and the non-alopecic occipital area of the same participant. A total of seven such paired samples were obtained. Four pairs were used for multiple omics analysis, and the remaining three pairs were used for tissue immunofluorescence (IF) validation.

### 2.2. RNA-Seq Analysis

Biopsy samples of full-thickness scalp were collected and promptly stored at −80 °C for later RNA sequencing and proteomic analyses. Total RNA isolation and library construction adhered to established protocols detailed in the Supplementary Method S1. Quality control was passed by all samples before RNA sequencing was executed.

### 2.3. Proteomics Analysis

Proteomic samples underwent labeling through Tandem Mass Tagging (TMT) among other quantitative techniques. Protein extraction, TMT labeling and quantification of all samples were performed by LC BioTechnology Co., Ltd. (Hangzhou, China). These proteins were then fractionated by Easy-nLC chromatography and subjected to mass spectrometry analysis using a Q Exactive Plus spectrometer (Thermo Fisher Scientific, San Jose, CA, USA). The MS/MS raw files were processed using MASCOT engine (Matrix Science, London, UK; version 2.6) embedded into Proteome Discoverer 2.2, and searched against the Uniprot database. More details are present in the Supplementary Method S1.

### 2.4. Differential Expression and Bioinformatics Analysis

Differential analysis of transcriptomic and proteomic data expression abundances was conducted using the DESeq2 package (version 1.44.0) in R (version 4.2.3). Differential expression proteins/genes/miRNAs (DEPs/DERs/DEmiRNAs) were identified using fold change (FC) and *p*-values, with significance set at *p* < 0.05. The criteria for selecting DEPs included: *p* < 0.05 and FC > 1.2 for upregulation; *p* < 0.05 and FC < 1/1.2 for downregulation. For DERs/DEmiRNAs, the criteria were: *p* < 0.05 and FC > 2 for upregulation; *p* < 0.05 and FC < 0.5 for downregulation. Significantly different miRNAs were predicted for target genes using TargetScan (v5.0) and miRanda (v3.3a) software. The selection threshold for target genes was TargetScan_score ≥ 50 & miranda_Energy < −20. Enrichment analyses for DEPs/DERs/DEmiRNAs-mRNA were performed using the “clusterProfiler” R package (V4.14.3), focusing on Gene Ontology (GO) and Kyoto Encyclopedia of Genes and Genomes (KEGG) pathways. Venn diagrams were employed to identify genes/proteins commonly upregulated and downregulated in AGA among both DERs and DEPs. These genes were analyzed for protein–protein interactions (PPI) using STRING (http://string-db.org/) and visualized with Cytoscape 3.9.1, employing the Matthews Correlation Coefficient (MCC) algorithm to select hub genes. Gene Set Enrichment Analysis (GSEA) was conducted to jointly validate the activation status of pathways in the transcriptome and the proteomics dataset. Target relationships between mRNA and miRNA were again predicted using TargetScan (v5.0) and miRanda (v3.3a). miRNA-lncRNA pairs were predicted based on the starBase website with clipExpNum > 5, removing duplicates to construct a ceRNA network based on mRNA_miRNA and mRNA_miRNA_lncRNA interactions.

### 2.5. Cell Culture

Human primary outer root sheath cells (ORSCs) were isolated from the scalp tissue of healthy donors. Subsequently, following the supplier’s guidelines, they were cultured and passaged to the third generation in primary epithelial cell culture medium (Zhejiang Meisen Cell Technology Co., Ltd., Jinhua, China, CTCC-001-PriMed) under conditions of 37 °C constant temperature, appropriate humidity, and 5% CO_2_ environment [12]. These cells were enzymatically detached using 0.25% trypsin at 37 °C for approximately 3 min and then passaged into 12-well plates at a seeding density of 60% confluence.

### 2.6. Dual-Luciferase Reporter Assay

ORSCs were co-transfected with luciferase reporter plasmids containing the wild-type (WT) or mutant (MUT) binding sites of the 3′UTR of NC *ACAA1* and miR-1343-3p mimics or mimic negative controls (NC) using GP-transfect-Mate (GenePharma, Shanghai, China). The luciferase reporter gene plasmids were constructed by GenePharma Technology (Shanghai, China). Twenty-four hours post-transfection, cells were lysed and the Renilla luciferase activity was compared using a Dual Luciferase Reporter Assay Kit (GenePharma) and a multifunctional microplate reader (BioTek, Winooski, VT, USA). The miR-1343a-3p mimics and mimic NC were provided by GenePharma, with the following sequences: the sequence of miR-1343-3p mimics is 5′-CUCCUGGGGCCCGCACUCUCGC-3′, and the sequence of mimics NC is 5′-UUCUCCGAACGUGUCACGUTT-3′. Additionally, the sequence of miR-3609_R-2 mimics is 5′-CAAAGUGAUGAGUAAUACUGGC-3′, and the sequence of mimics NC is 5′-UUCUCCGAACGUGUCACGUTT-3′.

### 2.7. Tissue Immunofluorescence

Scalp tissues were fixed in 4% Paraformaldehyde, embedded in paraffin, and cut into 5 μm sections. After deparaffinization and antigen retrieval, sections were blocked and incubated with primary antibodies ACAA1 (1:50, 12319-2-AP, Proteintech, Rosemont, IL, USA), IDH1 (1:500, ab256557, Abcam, Cambridge, UK), PEX3 (1:200, 30424-1-AP, Proteintech), and DBI (1:4000, ab317823, Abcam) at 4 °C overnight. Following washing, sections were incubated with secondary antibodies for 1 h at room temperature. Nuclei were counterstained with DAPI (HKI0005, Haoke Biotechnology, Hangzhou, China). Images were acquired using fluorescence microscopy, and the mean fluorescence intensity (Mean) from three random fields per sample was quantified via Fiji/ImageJ (V2.3.0) for statistical analysis.

### 2.8. Statistical Analysis

The bioinformatics analysis was performed using R (version 4.2.3). Differential expression analysis for transcriptomics was conducted using DESeq2 with the Wald test, while proteomics analysis was based on *t*-tests. GO and KEGG enrichment analyses were performed using hypergeometric distribution tests, as detailed in Section 2.4 (Differential Expression and Bioinformatics Analysis). GO results were visualized based on the top 20 terms ranked by *p*-value significance. Immunofluorescence and luciferase reporter assays were analyzed using GraphPad Prism 8.0 (GraphPad Software, San Diego, CA, USA) with unpaired, two-tailed Student’s *t*-tests, and a *p*-value < 0.05 was considered statistically significant.

## 3. Results

### 3.1. Identification of DEPs, DERs, and DEmiRNA

**Figure 1:**
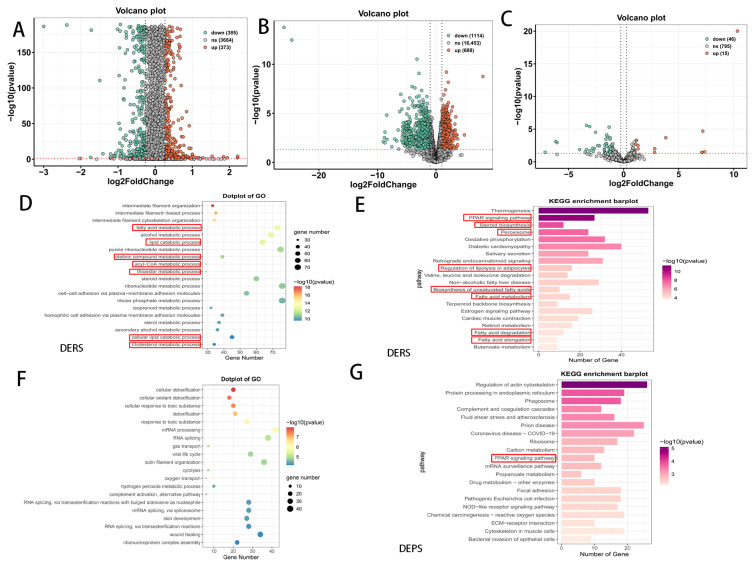
The mRNA and protein levels consistently indicated significant involvement of lipid metabolism-related pathways in AGA. (**A**) Volcano plot of DEPs and corresponding numbers. (**B**) Volcano plot of DERs and corresponding numbers. (**C**) Volcano plot of DEmiRNAs and corresponding numbers. (**D**) GO enrichment analysis of DERs revealed various lipid metabolic processes were notably enriched (highlighted with red rectangles). (**E**) KEGG enrichment analysis for DERs showed pathways such as the estrogen signaling pathway, and lipid metabolic signaling pathway (highlighted with red rectangles). (**F**) GO enrichment analysis of DEPs. (**G**) KEGG enrichment analysis for DEPs supported the significant enrichment of the PPAR signaling pathway (highlighted with red rectangles). The red dashed line in (**A**–**C**) represents −log_10_(*p*-value) = 1.3 (*p* < 0.05).

To elucidate the underlying mechanisms and identify potential therapeutic targets for AGA, differential expression analyses were conducted between the bald and non-bald groups across protein, mRNA, and miRNA levels (Figure 1). A total of 758 DEPs were identified, with 373 upregulated and 385 downregulated in the alopecia group compared to the non-alopecia group (Figure 1A, Table S1). Additionally, 1802 DERs were identified, comprising 688 upregulated and 1114 downregulated (Figure 1B, Table S2), and 61 DEmiRNAs, with 15 upregulated and 46 downregulated (Figure 1C, Table S3). Following the prediction of miRNA target genes, 9461 miRNA-mRNA interactions were identified (Table S4). DEPs and DERs were subsequently analyzed for enrichment in GO and KEGG pathways. The GO enrichment analysis of DERs revealed significant enrichment in processes related to cytoskeletal regulation pertinent to AGA pathogenesis, such as intermediate filament organization and cytoskeleton organization. Additionally, various lipid metabolic processes were notably enriched, including fatty acid metabolic processes, lipid catabolic process, acyl-CoA metabolic process, steroid metabolic process, sterol metabolic process, cellular lipid catabolic process, and cholesterol metabolic process (Figure 1D, Table S5). In the GO enrichment analysis for DEPs, lipid-related metabolic processes were significantly enriched (*p* < 0.02) (Figure 1E, Table 1 and Table S6), including fatty acid binding, protein-lipid complex, fatty acid beta-oxidation, etc. The KEGG enrichment analysis for DERs showed significant involvement of pathways such as the estrogen signaling pathway, PPAR signaling pathway, steroid biosynthesis, and fatty acid metabolic processes, among others (Figure 1F, Table S7). The KEGG enrichment analysis for DEPs further supported the significant enrichment of the PPAR signaling pathway (Figure 1G, Table S8). In summary, the GO and KEGG enrichment analyses at the mRNA and protein levels consistently indicated significant involvement of fatty-related metabolic pathways in AGA.

### 3.2. Combined Analysis of Co-Expressed mRNA and Protein

**Figure 2:**
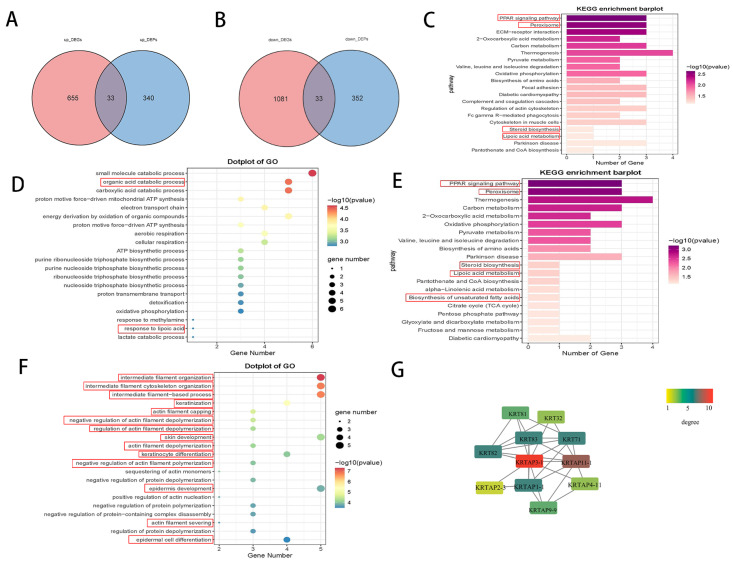
Combined analysis of co-expressed mRNA and protein suggesting while lipid metabolism-related pathways are activated, keratin-related metabolic processes are inhibited. (**A**) 33 genes that are simultaneously upregulated at both the DEG and DEP levels (**B**) 33 genes that are simultaneously downregulated at both the DEG and DEP levels. (**C**) KEGG enrichment analysis of co-expressed 66 genes/proteins revealed enrichment in lipid metabolism-related pathways (highlighted with red rectangles) Further enrichment analysis was performed separately for the upregulated and downregulated genes. (**D**) GO terms for the upregulated genes showed enrichment in organic acid catabolic processes and response to lipoic acid (highlighted with red rectangles). (**E**) KEGG pathway analysis for the upregulated genes revealed pathways such as the PPAR signaling pathway, etc., which all associated with fatty acid metabolism (highlighted with red rectangles). (**F**) GO analysis of the downregulated genes highlighted enrichment in hair loss pathways related to intermediate filaments, keratin, and epithelial development (highlighted with red rectangles). (**G**) The protein–protein interaction network related to the downregulated genes.

**Figure 3:**
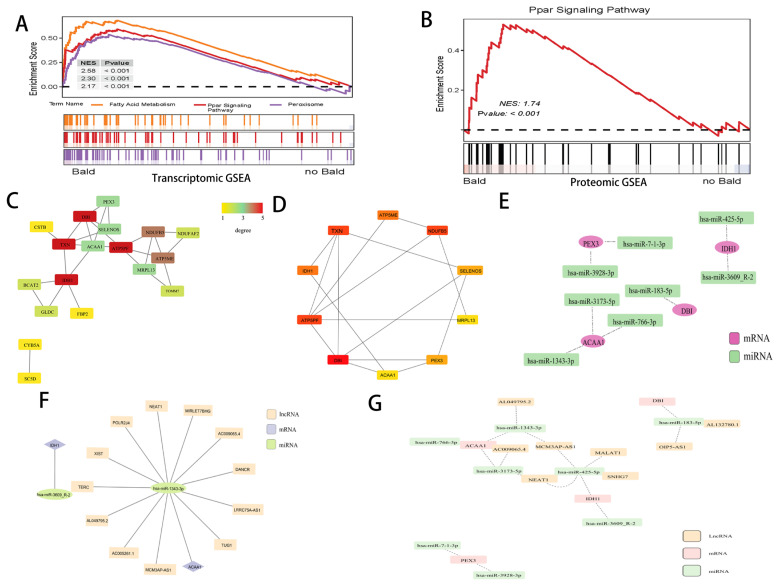
Candidate genes screen, ceRNA Networks construction and miRNA screen. (**A**) Transcriptomic GSEA of lipid-related pathways in scalp tissues. NES: Normalized Enrichment Score. (**B**) Proteomic GSEA confirms specific activation of PPAR signaling pathway in Bald group. NES: Normalized Enrichment Score. (**C**) The protein–protein interaction network related to the upregulated genes. (**D**) Top 10 hub genes were identified using the MCC-based algorithm of the cytoHubba plug-in in Cytoscape. (**E**) miRNA-mRNA interaction network of Candidate genes. (**F**) mRNA-miRNA-lncRNA ceRNA network of Candidate genes. (**G**) mRNA-miRNA-lncRNA network featuring the two miRNAs, two mRNAs, and 12 lncRNAs indicates that *ACAA1* and hsa-miR-1343-3p play predominant roles in mechanisms.

An integrated analysis of mRNA and protein expression was conducted to identify genes/proteins significantly co-expressed at both levels. A total of 33 genes/proteins were found to be simultaneously upregulated, and 33 were simultaneously downregulated (Figure 2A,B). Pathway enrichment analysis of these 66 genes revealed significant enrichment in lipid metabolism-related pathways, including the PPAR signaling pathway, peroxisome, steroid biosynthesis, and lipoic acid metabolism (Figure 2C, Table S9). Further enrichment analysis was performed separately for the upregulated and downregulated genes. For the upregulated genes, GO terms showed significant enrichment in processes such as organic acid catabolic processes and response to lipoic acid (Figure 2D, Table S10). KEGG pathway analysis revealed enrichment in pathways such as the PPAR signaling pathway, peroxisome, steroid biosynthesis, lipoic acid metabolism, and biosynthesis of unsaturated fatty acids—all associated with fatty acid metabolism (Figure 2E, Table S11). For the downregulated genes, GO analysis highlighted enrichment in pathways related to intermediate filament organization, keratinization, keratinocyte differentiation, epidermis development, and other processes related to intermediate filaments, keratin, and epithelial development (Figure 2F, Table S12). The KRTAP gene family (Keratin-Associated Protein gene family) serves as the core molecular foundation for the formation and functional exertion of the hair shaft structure. Keratin-associated proteins (KAPs) are key structural components in hair fibers. Our PPI analysis results identified a cluster among the 33 downregulated genes, which includes members related to the KRTAP gene family (such as *KRTAP3-1*, *KRTAP11-1*, *KRTAP2-3*, *KRTAP1-1*, *KRTAP4-11*, and *KRTAP9-9*) as well as KRT genes (for example, *KRT81*, *KRT83*, *KRT71*, and *KRT82*) (Figure 2G). GSEA suggested the activation of some lipid metabolism-related pathways, including fatty acid metabolism, PPAR signaling pathway, and peroxisome pathway (Figure 3A–C).

### 3.3. Screening Candidate Genes

In the prior analysis, a link was established between lipid metabolism and AGA, characterized by upregulated genes. To further probe this association, a PPI network was constructed for these genes using the STRING database. This network consisted of 18 proteins and 26 nodes (Figure 3D). The top 10 hub genes—*DBI*, *ATP5PF*, *TXN*, *NDUFB5*, *IDH1*, *ATP5ME*, *SELENOS*, *PEX3*, *MRPL13*, and *ACAA1*—were identified using the MCC-based algorithm of the cytoHubba plug-in in Cytoscape (Figure 3E). Pathway enrichment analysis (*p* < 0.05) indicated that these co-upregulated genes participate in lipid metabolism-related pathways, notably the PPAR signaling pathway, peroxisome, Steroid biosynthesis and Lipoic acid metabolism, all significant in the KEGG pathway analysis. By overlapping the top 10 hub genes with genes enriched in lipid metabolism pathways, four candidate genes were pinpointed as central to AGA: *DBI*, *ACAA1*, *IDH1*, and *PEX3* (Table 2). These genes are critical at the nexus of differential expression, hub gene status, and lipid metabolism.

### 3.4. Constructing ceRNA Networks

To explore the regulatory relationships between mRNA and miRNA, the TargetScan and miRanda databases were utilized to predict interactions involving the four core genes. Non-human miRNAs were excluded. A total of eight miRNAs were pinpointed: hsa-miR-183-5p, hsa-miR-3609_R-2, hsa-miR-425-5p, hsa-miR-3928-3p, hsa-miR-7-1-3p, hsa-miR-1343-3p, hsa-miR-3173-5p, and hsa-miR-766-3p, and were employed to construct the miRNA-mRNA interaction network (Figure 3F). Furthermore, these eight miRNAs were applied to predict interactions with long non-coding RNAs (lncRNAs) through the starBase database. miRNA-lncRNA pairs with clipExpNum > 10 were selected, leading to the identification of eight lncRNAs: AL049795.2, MCM3AP-AS1, AL132780.1, OIP5-AS1, NEAT1, AC009065.4, SNHG7, and MALAT1. Subsequently, an mRNA-miRNA-lncRNA ceRNA network was constructed (Figure 3G), elucidating the intricate regulatory mechanisms in AGA.

### 3.5. Screening miRNA

Moreover, two miRNAs—hsa-miR-3609_R-2 and hsa-miR-1343-3p—were identified by intersecting the eight predicted miRNAs with the 61 differentially expressed miRNAs (DEmiRNAs) derived from sequencing. These miRNAs correspond to two mRNAs of the four hub genes: *IDH1* and *ACAA1*. Using the starBase database, lncRNAs targeting these miRNAs were predicted, and miRNA-lncRNA pairs with clipExpNum > 5 were selected, resulting in the identification of 12 lncRNAs. An mRNA-miRNA-lncRNA network was subsequently constructed, featuring the two miRNAs, two mRNAs, and 12 lncRNAs (Figure 3F). This network indicates that *ACAA1* and hsa-miR-1343-3p play predominant roles in the regulatory mechanisms.

### 3.6. Experimental Findings

**Figure 4:**
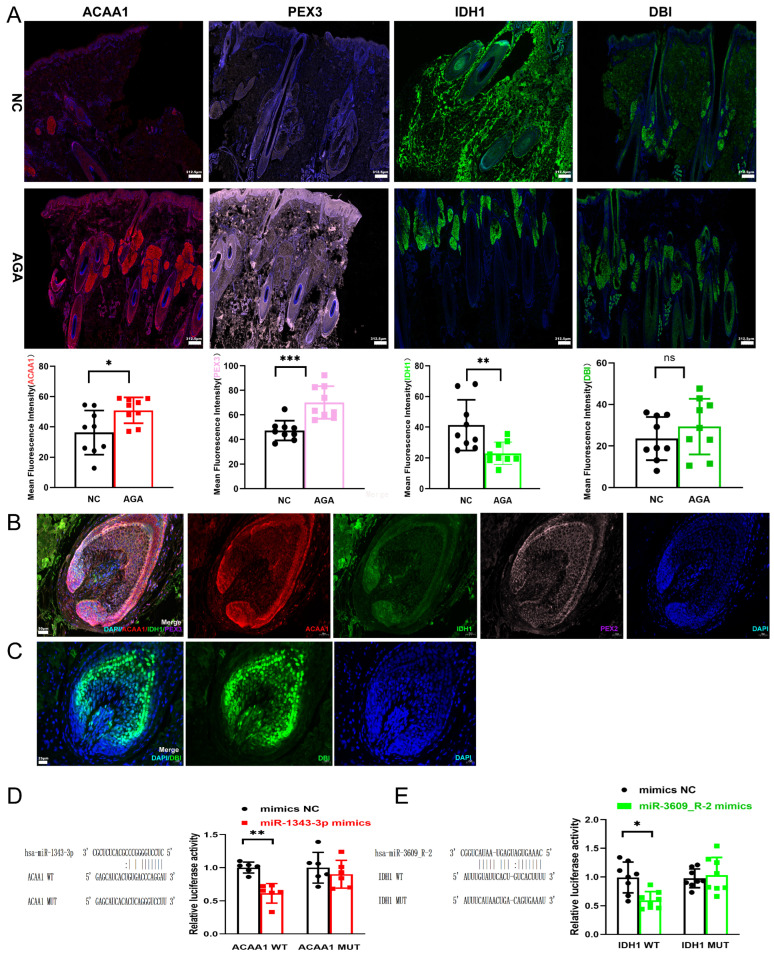
The expression of ACAA1, IDH1, PEX3 and DBI in human scalp and the results of dual-luciferase reporter assay. (**A**) Validation of the expression of ACAA1, IDH1, PEX3 and DBI involved in the lipid metabolism in human scalp tissue from AGA and NC. Quantitative analysis reveals a significant upregulation of ACAA1and PEX3 in scalp tissues from AGA patients compared to NC. In contrasts, IDH1 shows downregulation, while DBI exhibits no significant change. (**B**) Localization in the hair bulb revealed expression of ACAA1, IDH1, and PEX3 in hair follicle cells, including dermal papilla cells and ORSCs. (**C**) Localization in the hair bulb revealed expression of DBI in dermal papilla cells without expression of DBI in ORSCs. (**D**) Binding sites and mutation sites of *ACAA1* via miR-1343-3p. In the WT group, co-transfection with hsa-miR-1343-3p mimics and the *ACAA1* WT plasmid resulted in a significant decrease in luciferase activity compared to the mimics NC group, with no change observed in the MUT group. (**E**) Binding sites and mutation sites of *IDH1* via miR-3609_R-2. In the WT group, co-transfection with hsa-miR-3609_R-2 mimics and the *IDH1* WT plasmid resulted in a significant decrease in luciferase activity compared to the mimics NC group, with no change observed in the MUT group. AGA, androgenetic alopecia; NC, normal controls; ORSCs, outer root sheath cells; WT, the wild-type reporter plasmid; MUT, the mutant reporter plasmid; mimics NC, negative control plasmid; miR-3609_R-2 mimics, overexpressed miR-3609_R-2 plasmid; miR-1343-3p mimics, overexpressed miR-1343-3p plasmid. Scale bars, 312.5 μm. * *p* < 0.05; ** *p* < 0.01; *** *p* < 0.001; ns *p* > 0.05.

Tissue IF analysis in scalp tissues from AGA patients suggested altered protein levels in AGA patients compared to NC subjects. Specifically, the mean fluorescence intensity for ACAA1 and PEX3 was observed to be significantly higher in AGA tissues (*p* = 0.0188 and *p* = 0.0004, respectively, Figure 4A), a finding consistent with our earlier analyses. Conversely, the signal for IDH1 was significantly lower in the AGA group (*p* = 0.0075, Figure 4A), while DBI intensity levels showed no significant difference (Figure 4A). Localization analysis in the hair bulb indicated that ACAA1, IDH1, and PEX3 were expressed in hair follicle cells, including dermal papilla cells and outer root sheath cells (ORSCs) (Figure 4B). In contrast, expression of DBI was not detected in ORSCs (Figure 4C). These observations in ORSCs are particularly noteworthy, as these cells’ proliferative and migratory capabilities are crucial for hair follicle regeneration and are implicated in AGA pathogenesis. The “mimic” is an artificially introduced miRNA substitute simulating endogenous miRNA’s inhibitory effects. In the WT group, co-transfection with hsa-miR-1343-3p mimics and the *ACAA1* WT plasmid resulted in a significant decrease in luciferase activity compared to the mimics NC group (*p* = 0.059, Figure 4D), with no change observed in the MUT group. This indicates that hsa-miR-1343-3p directly targets the 3′UTR of *ACAA1*, thereby confirming *ACAA1* as a direct target gene of miR-1343-3p. Similarly, we also confirmed the targeting of *IDH1* by hsa-miR-3609_R-2, which showed a significant difference in expression pattern (*p* = 0.0105, Figure 4E) with a significant different expression pattern.

## 4. Discussion

Although direct foundational research linking lipid metabolism to AGA is relatively limited, existing evidence indicates that lipids play a crucial role in hair growth by providing not only energy but also structural components [13]. Studies show that, during the anagen phase, dermal adipocytes shift from lipogenesis to lipophagy and lipolysis, releasing cholesterol that is absorbed by hair follicles. This cholesterol promotes steroidogenesis and facilitates the transition from anagen to catagen [6]. Lipid metabolism may also influence hair growth and cycling by altering the microenvironment of hair follicle stem cells and bulge cells [6]. Lipid metabolites, including steroid hormones and fatty acids, are involved in signaling and senescence processes in hair follicle stem cells [6,14]. High-fat diets lead to lipid droplet accumulation in hair follicle stem cells, activating the NF-κB pathway, inhibiting the Sonic Hedgehog (SHH) pathway, and impairing stem cell renewal, thereby accelerating follicle miniaturization and hair loss [15]. In our study, GO and KEGG analyses of DERs and DEPs revealed significant enrichment in lipid metabolism pathways. Subsequent co-expression analysis confirmed the upregulation of numerous genes at both mRNA and protein levels, primarily related to lipid metabolism, including the PPAR signaling pathway, steroid biosynthesis, and fatty acid metabolism.

Furthermore, our GSEA and KEGG enrichment analyses of DERs, DEPs, and upregulated genes consistently indicated significant activation of the PPAR signaling pathway. The role of the PPAR signaling pathway in AGA appears to involve complex mechanisms of activation and inhibition. On one hand, PGC1α, which acts as a co-activator of PPARγ, has been associated with hair miniaturization due to its upregulation. PPARγ, observed in the hair follicles of AGA patients, is known to inhibit keratinocyte proliferation and promote terminal differentiation [16]. On the other hand, there is an interaction between PPARγ signaling and androgen receptor signaling, which affects androgen synthesis [17]. The deletion of PPARγ in hair follicle stem cells has been linked to the pathogenesis of cicatricial alopecia. Agonists of PPARγ may enhance the survival of hair follicle stem cells by reducing inflammation and modulating lipid metabolism, potentially preventing follicular miniaturization and hair loss [18,19]. Additionally, PPARα plays a role in influencing the development of atherosclerosis and cardiovascular diseases by regulating blood lipid levels and exerting anti-inflammatory effects, mechanisms that may overlap with the pathophysiology of alopecia [18].

Dysregulation of genes related to lipid metabolism can impair hair follicle health through multiple pathways. For instance, reduced DNA methylation and decreased Elovl2 expression disrupt lipid metabolism, interfering with endoplasmic reticulum and mitochondrial function, accelerating senescence, and leading to hair loss [19,20]. Stearoyl-CoA desaturase 1 (SCD1), a key enzyme in fatty acid desaturation, is essential for Wnt3a protein palmitoylation, which regulates the hair growth cycle. SCD1 deficiency results in prolonged follicle elongation, hindering bulge structure formation and disrupting hair cycling [21,22]. ABCA5, another critical gene in lipid metabolism, regulates cholesterol homeostasis in hair follicle keratinocytes; impaired ABCA5 activity negatively affects hair growth [23].

Our study identified four genes—*ACAA1*, *IDH1*, *PEX3*, and *DBI*—as playing key roles in differential expression, hub gene status, and their association with lipid metabolism. Using these genes, we constructed miRNA-mRNA and ceRNA interaction networks, further elucidating their complex regulatory mechanisms in AGA. Notably, integrative analysis revealed concordant upregulation of *ACAA1* and *IDH1* at the transcriptional (mRNA), translational (protein), and post-transcriptional (microRNA) regulatory tiers. Network analysis and DLR assays confirmed that *ACAA1* is targeted by hsa-miR-1343-3p, while *IDH1* is targeted by hsa-miR-3609_R-2. hsa-miR-1343-3p has multiple target genes and has been reported to participate in energy metabolism, fatty acid metabolic reprogramming, and proliferation in tumor cells [24,25]. For example, it is involved in fatty acid metabolic reprogramming in triple-negative breast cancer, further emphasizing the role of fatty acid metabolism in AGA at the microRNA level [24]. Tissue IF analysis initially supported our prediction of upregulated ACAA1 expression in AGA. ACAA1, functionally linked to the PPAR signaling pathway, peroxisomes, and fatty acid metabolic processes, encodes a key peroxisomal thiolase that catalyzes the terminal step of fatty acid β-oxidation. This pathway supplies substrates to the tricarboxylic acid cycle and helps maintain cellular lipid homeostasis. Variants in ACAA1 may impair lysosomal function and autophagy, thereby disrupting lipid metabolism in hair follicle cells and compromising their growth and regenerative capacity [26]. Based on our analysis that ACAA1 is upregulated at both the transcript and protein levels in this study, we speculate that ACAA1 may play an important role in AGA by participating in PPAR signaling. Furthermore, IDH1 is a pivotal metabolic enzyme primarily located in the cytoplasm and peroxisomes. It catalyzes the conversion of isocitrate to α-ketoglutarate while simultaneously producing NADPH, a crucial cellular reductant essential for antioxidant defense mechanisms [27]. Fluctuations in IDH1 expression can alter NADPH levels, thereby affecting oxidative stress balance and fatty acid metabolism, both of which may affect AGA progression. In this study, we confirmed that hsa-miR-3609_R-2 targets *IDH1*; however, contrary to our expectations based on mRNA and network analyses (which suggested upregulation), tissue IF analysis showed decrease in IDH1 in mean fluorescence intensity.

In summary, this research provides preliminary evidence from a multi-omics perspective for the involvement of lipid metabolism and associated genes in AGA pathogenesis, with a particular focus on the targeting of ACAA1 by hsa-miR-1343-3p. These findings highlight the complex regulatory networks in lipid-related pathways and their potential role in hair follicle dysfunction. From a clinical perspective, nutritional management is a frequently overlooked yet crucial aspect in the prevention and treatment of AGA. Evidence suggests that a pro-inflammatory diet—particularly one rich in trans fats and saturated fats—may increase the risk of developing AGA by inducing metabolic syndrome [28]. Our research further indicates that dysregulated lipid metabolism, involving genes such as *ACAA1* and *PEX1*, is a key pathological feature of AGA. Therefore, dietary interventions aimed at optimizing systemic lipid status may represent a novel preventive or adjunctive therapeutic approach for AGA. Furthermore, the discovery of the hsa-miR-1343-3p/*ACAA1* regulatory axis provides a molecular target for treatment. By correcting the imbalance in lipid metabolism within hair follicles, this axis has the potential to halt or reverse the miniaturization process characteristic of AGA. However, our study has several limitations. A key strength is the paired-sample design, which controls for inter-individual variability. However, this approach is constrained by the intrinsic biological differences between the AGA-affected frontal scalp and the typically unaffected occipital control sites. The molecular changes we observed may therefore be a composite of true pathology and these inherent anatomical variations. Confirming these findings will require future studies that, despite the practical challenges, use the ideal control: frontal scalp tissue from healthy, non-balding individuals.

Additionally, although we have preliminarily explored the role of lipid metabolism in AGA from a multi-omics perspective, future studies will require in-depth lipidomic analyses to validate and expand these findings. The relatively small sample size also necessitates further validation of IDH1 expression in larger cohorts. Additional cellular and animal studies are warranted to substantiate how miRNAs regulate energy metabolism, proliferation, and differentiation of hair follicle cells by modulating lipid metabolism enzymes, thereby participating in the formation of the AGA state.

Finally, it is essential to clarify that our findings provide a molecular snapshot of the terminal state in AGA. For instance, the absence of keratins in balding samples depicts the endpoint—a miniaturized follicle—rather than the dynamic processes leading to it (Figure 2G). Thus, our results are primarily descriptive, elucidating “what” the follicle becomes, but not “how” or “why.” While these insights offer valuable biomarkers for advanced AGA, future research is imperative to unravel the causal mechanisms driving this transition, such as determining whether it represents the ultimate outcome of dihydrotestosterone influence.

## Figures and Tables

**Table 1:** GO enrichment analysis for DEPs. Lipid-related metabolic processes were significantly enriched, including fatty acid binding, protein-lipid complex, fatty acid beta-oxidation, etc.

ID	Description	GeneRatio	BgRatio	*p* Value	Count
GO:0005504	fatty acid binding	7/718	49/18,369	0.003	7
GO:0032994	protein-lipid complex	6/729	39/19,518	0.003	6
GO:0006635	fatty acid beta-oxidation	8/703	76/18,614	0.008	8
GO:0001676	long-chain fatty acid metabolic process	10/703	110/18,614	0.009	10
GO:0006636	unsaturated fatty acid biosynthetic process	6/703	52/18,614	0.01	6
GO:0010883	regulation of lipid storage	6/703	53/18,614	0.01	6
GO:0006631	fatty acid metabolic process	24/703	394/18,614	0.02	24
GO:0010884	positive regulation of lipid storage	4/703	27/18,614	0.02	4
GO:0009062	fatty acid catabolic process	9/703	105/18,614	0.02	9
GO:0031999	negative regulation of fatty acid beta-oxidation	2/703	6/18,614	0.02	2

GO, Gene Ontology; DEP, differential expressed proteins.

**Table 2:** Overlapping the top 10 candidate genes with genes enriched in lipid metabolism pathways. Four pivotal genes were pinpointed as central to AGA: *DBI*, *ACAA1*, *IDH1*, and *PEX3*.

ID	Description	*p* Value < 0.05	geneID	Count
hsa03320	PPAR signaling pathway	*p* < 0.001	*DBI*/*PLIN2*/*ACAA1*	3
hsa04146	Peroxisome	*p* < 0.001	*IDH1*/*PEX3*/*ACAA1*	3
hsa04714	Thermogenesis	0.002	*ATP5ME/ATP5PF*/*NDUFB5*/*NDUFAF2*	4
hsa01200	Carbon metabolism	0.002	*IDH1*/*GLDC*/*FBP2*	3
hsa01210	2-Oxocarboxylic acid metabolism	0.002	*IDH1*/*BCAT2*	2
hsa00190	Oxidative phosphorylation	0.003	*ATP5ME*/*ATP5PF*/*NDUFB5*	3
hsa00620	Pyruvate metabolism	0.005	*LDHD*/*ACYP2*	2
hsa00280	Valine, leucine and isoleucine degradation	0.005	*BCAT2*/*ACAA1*	2

## Data Availability

The data that support the findings of this study are available from the corresponding authors upon reasonable request. Informed consent was obtained from all subjects involved in the study.

## Supplementary Materials

The following supporting information can be downloaded at: https://www.mdpi.com/article/10.3390/biomedicines14010160/s1, Table S1. The list of differential expressed proteins. Table S2. The list of differential expressed mRNA. Table S3. The list of differential expressed microRNA. Table S4. The list of prediction of miRNA target genes. Table S5. The list of the GO enrichment analysis of DERs Table S6. The list of the GO enrichment analysis of DEPs. Table S7. The list of the KEGG enrichment analysis of DERs. Table S8. The list of the KEGG enrichment analysis of DEPs. Table S9. The list of the KEGG enrichment analysis of 66 co-expressed DER-DEPs. Table S10. The list of the GO enrichment analysis of 33 co-upregulated DER-DEPs. Table S11. The list of the KEGG enrichment analysis of 33 co-upregulated DER-DEPs. Table S12. The list of the GO enrichment analysis of 33 co-downregulated DER-DEPs. Supplementary Methods S1: Experimental Methods in Transcriptomics and Proteomics.
