# Supplementary material for: Integrated Multi-Omics Analysis Reveals Dysregulated Lipid Metabolism as a Novel Mechanism in Androgenetic Alopecia
Source: Biomedicines. 2026 Jan 12;14(1):160. doi: 10.3390/biomedicines14010160 (PMC12838848; doi:10.3390/biomedicines14010160)
Supplement: Supplementary file 1 [file biomedicines-14-00160-s001.zip › Supplementary Method S1.pdf]

Supplementary Methods S1. Experimental Methods in Transcriptomics and Proteomics

1. RNA extraction, RNA-sequencing and bioinformatics analyses

1.1 RNA extraction

Total RNA from scalp tissues were extracted using Trizol (Thermo Fisher) following the manufacturer's instructions. Extracted RNA was quantified using NanoDrop ND-1000 (NanoDrop, Wilmington, DE), and RNA integrity was assessed by Agilent Bioanalyzer 2100 with RIN number >7.0, and confirmed by electrophoresis with denaturing agarose gel.

1.2 Library preparation

All four bald biopsies and four non-bald biopsies from four AGA patients were subjected to RNA-sequencing. RNA-seq library preparation was performed with 2μg of RNA per sample. Sequencing libraries of mRNA were performed on the illumina Novaseq 6000 platform by LC Bio Technology CO., Ltd (Hangzhou, China). Sequencing was performed on Illumina Hiseq 2500 platform by LC Bio Technology CO., Ltd (Hangzhou, China).

1.3 Bioinformatics analysis of mRNAs

Fastp software (https://github.com/OpenGene/fastp) were used to remove the reads that contained adaptor contamination, low quality bases and undetermined bases with default parameter. Then sequence quality was also verified using Fastp. We used HISAT2 (https://ccb.jhu.edu/software/hisat2) to map reads to the human reference. The mapped reads of each sample were assembled using StringTie (https://ccb.jhu.edu/software/stringtie) with default parameters. Then, all transcriptomes from all samples were merged to reconstruct a comprehensive transcriptome using gffcompare (https://github.com/gpertea/gffcompare/). After the final transcriptome was generated, StringTie and was used to estimate the expression levels of all transcripts. StringTie was used to perform expression level for mRNAs by calculating FPKM (FPKM = [total_exon_fragments / mapped_reads(millions) × exon_length(kB)]).

2 Protein extraction, TMT-labeled proteomics, LC-MS/MS Analysis and bioinformatics analyses

2.1 Protein extraction, TMT labeling and quantification

Protein extraction, TMT labeling and quantification of all samples were performed by LC Bio Technology CO., Ltd (Hangzhou, China). Briefly, 100μg peptide mixture of each sample was labeled using TMT reagent according to the manufacturer's instructions (Thermo Fisher Scientific), and fractionated by reversed phase (RP) chromatography using the Agilent 1260 infinity II HPLC.

2.2 LC-MS/MS Analysis

Proteomics sequencing services were provided by LC Bio Technology CO., Ltd (Hangzhou, China). Each fraction was injected for nanoLC-MS/MS analysis on a Q

Exactive plus mass spectrometer (Thermo Fisher Scientific). Then the MS/MS raw files were processed using MASCOT engine (Matrix Science, London, UK; version 2.6) embedded into Proteome Discoverer 2.2, and searched against the Uniprot database.
